# The first steps in the development of a cancer-specific patient-reported experience measure item bank (PREM-item bank): towards dynamic evaluation of experiences

**DOI:** 10.1007/s00520-023-08266-5

**Published:** 2024-01-12

**Authors:** Kira S. van Hof, Karolijn Dulfer, Aniel Sewnaik, Robert J. Baatenburg de Jong, Marinella P. J. Offerman

**Affiliations:** 1 Department of Otorhinolaryngology and Head and Neck Surgery, Erasmus MC Cancer Institute, University Medical Center Rotterdam, Rotterdam, The Netherlands; 2 Department of Neonatal & Pediatric Intensive Care, Division of Pediatric Intensive Care, Erasmus MC-Sophia Children’s Hospital, Rotterdam, The Netherlands

**Keywords:** Patient-reported experience measure, Item bank, Head and neck cancer, Patient journey

## Abstract

**Objective:**

Since the implementation of value-based healthcare, there has been a growing emphasis on utilizing patient-reported experience measures (PREMs) to enhance the quality of care. However, the current PREMs are primarily generic and static, whereas healthcare is constantly evolving and encompasses a wide variety of aspects that impact care quality. To continuously improve care requires a dynamic PREM. The aim of this study was to propose an item bank for the establishment of a dynamic and care-specific patient-reported evaluation.

**Methods:**

In co-creation with patients, a mixed methods study was conducted involving: (1) an explorative review of the literature, (2) a focus group analysis with (ex-)patients, (3) qualitative analyses to formulate themes, and (4) a quantitative selection of items by patients and experts through prioritization.

**Results:**

Eight existing PREMs were evaluated. After removing duplicates, 141 items were identified. Through qualitative analyses of the focus group in which the patient journey was discussed, eight themes were formulated: “Organization of healthcare,” “Competence of healthcare professionals,” “Communication,” “Information & services,” “Patient empowerment,” “Continuity & informal care,” “Environment,” and “Technology.” Seven patients and eleven professionals were asked to prioritize what they considered the most important items. From this, an item bank with 76 items was proposed.

**Conclusion:**

In collaboration with patients and healthcare professionals, we have proposed a PREM-item bank to evaluate the experiences of patients’ receiving cancer care in an outpatient clinic. This item bank is the first step to dynamically assess the quality of cancer care provided in an outpatient setting.

**Supplementary Information:**

The online version contains supplementary material available at 10.1007/s00520-023-08266-5.

## Introduction

In the Netherlands, approximately 120,000 patients are diagnosed with cancer every year [1]. These patients often undergo a long and intensive healthcare trajectory from the moment of diagnosis through to long-term follow-up. Patients’ opinions about the care provided are an important quality measure as they can offer different perspectives and provide insights into aspects of healthcare that professionals are unaware of. Consequently, there is increasing attention given to the use of patient-reported experience measures (PREMs) in seeking to improve quality as part of value-based healthcare (VBHC) [2–4]. In the Netherlands, most tertiary hospitals measure patients’ experiences with the Picker Institute’s patient experience questionnaire [5]. This experience measure is based on “Picker’s principles of patient-centered care” and determines generic experiences of the care process using topics such as experience with “waiting time” and “friendliness of personnel” [5]. These generic PREMs are convenient for general quality improvements at the hospital level or for integrated care. Since the delivered care and logistic processes will differ between departments within a hospital (for example in “the use of volunteers on the diagnostic day” or “the use of prediction models to inform patients in decision making”), patients’ experiences with such care-specific processes are useful for improving quality at the department level [3].

Although there are potential advantages, the effectiveness of routine PREM assessments in clinical practice in enhancing healthcare services is uncertain as the evidence is inconclusive [6–9]. These inconsistent findings may be due to heterogeneity in the PREMs used. Healthcare is dynamic and contains a wide range of aspects that contribute to quality of care. To further enhance the healthcare process within a department, it is important to respond to opportunities for improvement identified through the PREM data. At a certain moment, when the items in the PREM are fully satisfied, it is no longer possible to further improve on these items and it is no longer useful to assess them. A more dynamic approach would offer the opportunity to switch to including other items covering another or new aspects of the healthcare process in seeking continuous improvement.

Based on this idea, we have developed a PREM-item bank for improving cancer-specific VBHC. This PREM-item bank complements the generic PREM (Picker) by providing opportunities to add specific or detailed questions about care experiences. This PREM item bank can be dynamically used to measure patients’ experiences with their care at the outpatient clinic.

## Methods

**Fig. 1:**
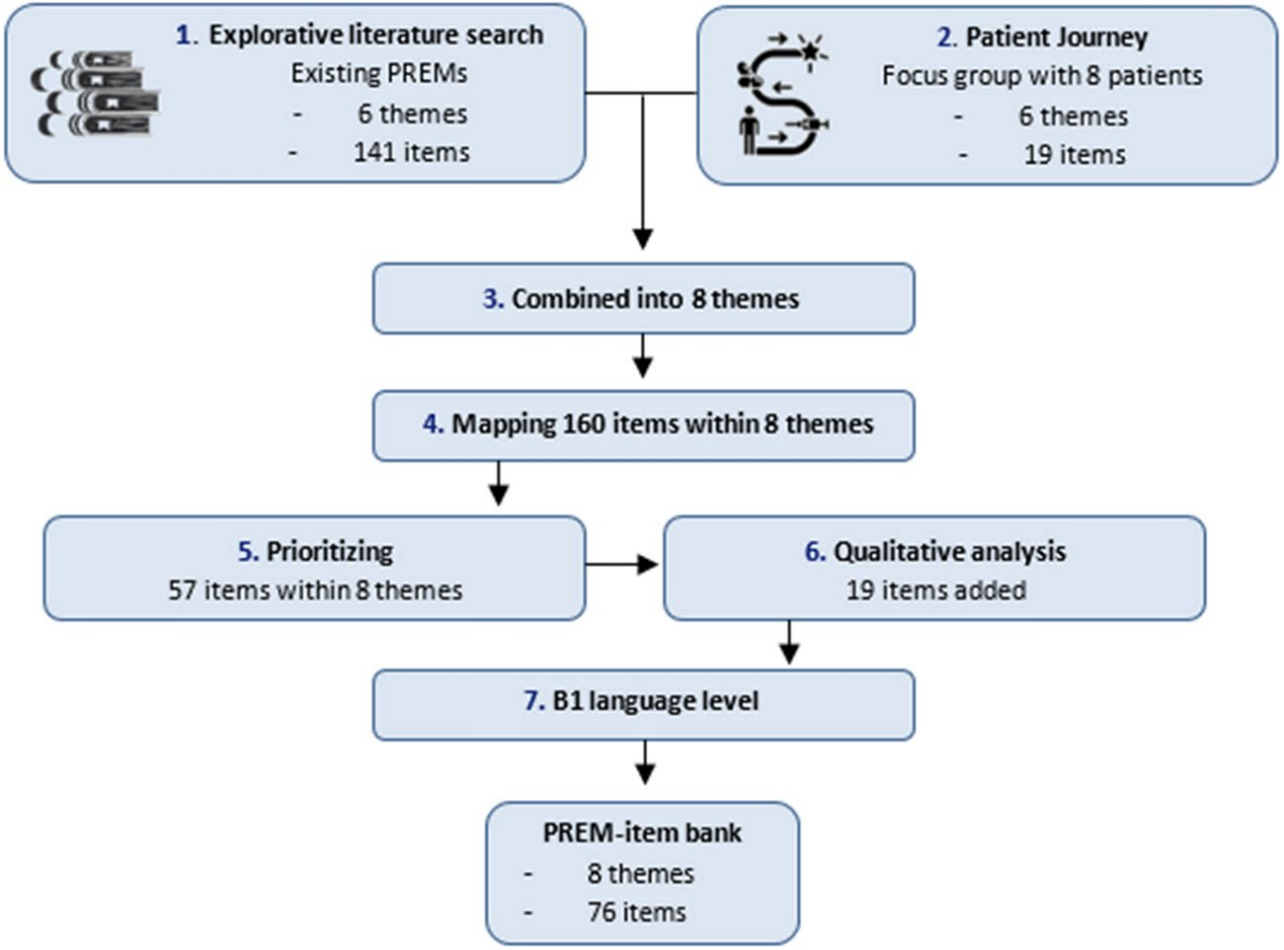
Development of the PREM-item bank. PREM, patient-reported experience measure; B1 language level, intermediate level

In a co-creation process with patients, we took the following steps to create the PREM-item bank: (1) a literature search to identify existing PREMs, (2) the identification of themes through focus group discussions in an approach based on patient journeys, (3) combining themes from the literature and themes from the qualitative analysis, (4) mapping by healthcare professionals of items within themes, (5) prioritizing the items seen as most important by patients and by healthcare professionals, and (6) rewriting items in a language understandable for most people (level B1) (Fig. 1).

### 1. Explorative literature search

An explorative literature search was performed using the PubMed, Embase, and Medline databases in August 2019 to obtain details of available generic and cancer-specific PREMs. For the search strategy, the words “patient reported experience measure” OR “experience measure” AND “cancer” were used. Articles were included when they described a PREM validated in the Dutch language that contained items assessing experience with the healthcare process covering both emotional and cognitive evaluation of the delivered care. We included generic and cancer-specific PREMs. Exclusion criteria were studies describing patient-reported outcomes measures (PROMs) and other questionnaires that did not include any patient-reported experience measures.

### 2a. Patient journey: focus group

**Fig. 2:**
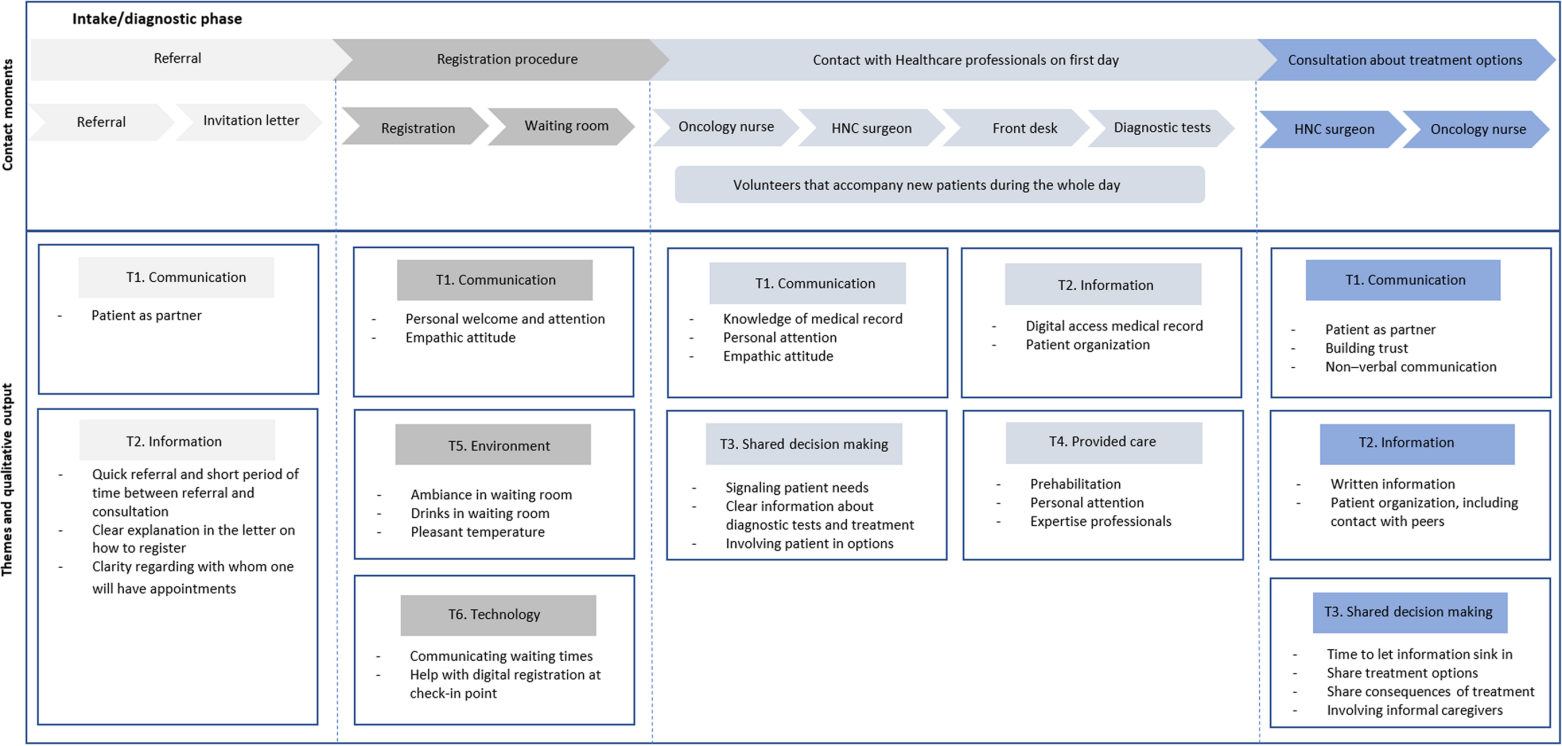
Contact moments in the diagnostic phase of the patient journey for HNC patients. HNC, head and neck cancer

**Fig. 3:**
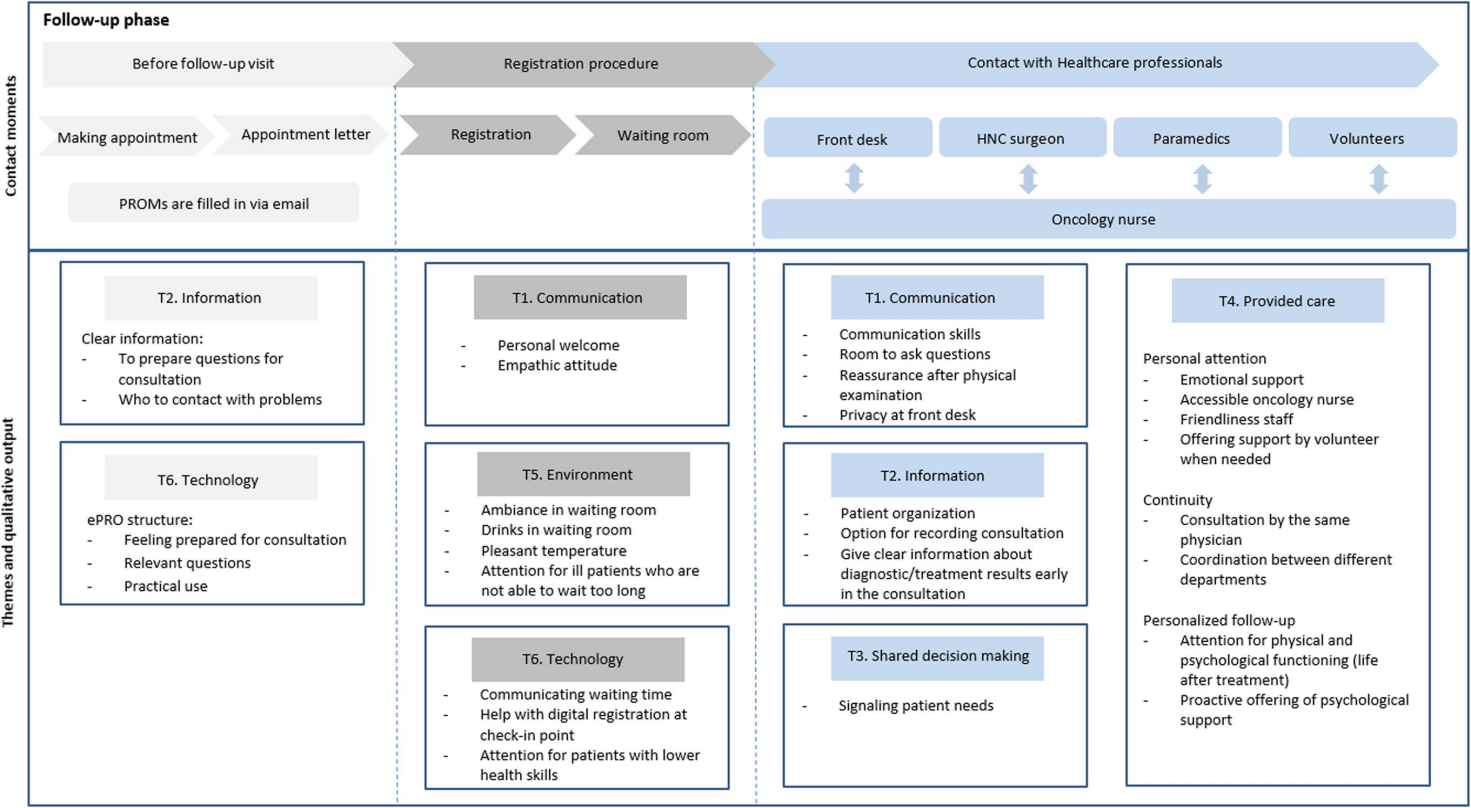
Contact moments in the follow-up phase of the patient journey for HNC patients. HNC, head and neck cancer; PROMs, patient-reported outcome measure; ePRO structure, electronic patient-reported outcome structure

Together with patients and healthcare professionals, we gathered relevant themes for the PREM-item bank. We asked (ex-) patients (*n* = 8) and healthcare professionals (*n* = 3) to participate in a focus group meeting during which a “patient journey of outpatient clinic experiences” was evaluated (Figs. 2 and 3). From our previous experience with focus groups and qualitative research, we know that a group size of 6–10 participants is ideal for ensuring a trusted environment and that everyone has a chance to speak, while also obtaining diverse input from different individuals. Creating a patient journey is a method to obtain the patient’s perspective on all (possible) activities and contact moments between patients and healthcare professionals. We focused on two moments in the outpatient clinic in this full patient journey: the diagnostic phase and the follow-up phase, as these specific contact periods contribute to how the total provided cancer care is experienced. The diagnostic phase was defined as the time between the moment of referral through to the consultation in which treatment options are discussed. Contact moments were grouped within four phases: referral, registration procedure, contact with healthcare professionals on intake to the hospital, and consultation about treatment options. The follow-up phase started with the first regular follow-up visit after treatment and ended at the last follow-up visit, five years after treatment. Contact moments were arranged in three phases: before the follow-up visit, during the registration procedure, and later contact with several healthcare professionals. Each possible contact moments was visualized and discussed separately. Patients were asked if they thought there were missing aspects in the proposed patient journey. Following this, all the steps were evaluated using the following questions: “What were positive experiences with the provided care?”, “What were negative experiences with the provided care?”, “What did you miss in the provided care?”, “Do you have suggestions to improve our care?”, and “Who or what was indispensable in the provided care?”. Data saturation was reached when no new themes were opted.

### 2b. Qualitative data analysis

An inductive approach for coding the data was followed by three researchers (MO, KD, and HW) who independently performed thematic analysis on the data. Consensus on the themes present was achieved through discussion, with verification by a fourth researcher (KH).

### 3. Combining themes from the literature and from the qualitative analysis

Three researchers (MO, KD, and HW) compared the themes identified from the literature with the themes from the focus group and formed eight new themes that covered different aspects of the intake or diagnostic phase and of the follow-up phase.

### 4. Mapping items within themes

After identifying suitable PREMs in step 1, all the individual items included in the PREMs were listed. Duplicates were removed. Items provided by the focus group that were not covered by the list of existing PREM questions were added. Following this, all the items were independently mapped on to the formulated themes by two senior researchers (MO and KD) and one junior researcher (HW). Consensus was achieved through later discussion.

### 5. Prioritizing

In order to select the most important items for each theme, a prioritization exercise was completed by both patients (*n* = 7) and 11 healthcare professionals (e.g., head and neck surgeons, psychologists, and oncology nurses). For themes with a fairly limited number of items (1, 2, 5, 7, and 8), a top-five list was compiled, while for more extensive themes (3, 4, and 6), a top-ten was generated. Items were weighted based on their ordering (i.e., first place = 5 and second place = 4). Final prioritization was based on the square root of the frequency of occurrence of an item multiplied by the weight of that item: $\sqrt{\left(\textrm{frequency}\ \textrm{of}\ \textrm{appearance}\ \textrm{x}\ \textrm{weight}\right)}$. The outcome was adjusted for group size such that both groups (patients and experts) had an equal influence on the outcome. Both healthcare professionals and patients were asked whether items were relevant, understandable, and whether important items were missing.

### 6. Qualitative analysis open comments

After the prioritization, three researchers (MO, KD, and HW) reviewed the open comments in which participants could formulate missing items. After discussion, the researchers reached a consensus on the items to be added when they had been mentioned multiple times and fit within the themes formulated from the previously conducted focus groups.

### 7. Rewriting items in Dutch language level B1 (intermediate level)

Next, all the items were rewritten using B1 language, i.e., put in a form that patients with limited health literacy could understand. Following the method applied with the Picker questionnaire, questions were partly repeated in the answer options to make them easier to understand [5]. The reformulated items were again sent to the participating patients to check for understandability.

## Results

### 1. Explorative literature search

Two previously validated generic PREMs (Consumer Quality Index (CQI) [10], Picker questionnaire [11]) plus five cancer-specific PREMs (FACE-Q head and neck [12], PREM VBHC head and neck [13], EORTC-INFO25 [14], and EORTC-COMU26 [15]) were included in the review. In addition, a PREM concerning the diagnostic day at the outpatient clinic constructed by the Department of Head and Neck Oncology of Erasmus Medical Center was included [16]. After removing duplications, 141 items were identified from the above instruments (Table 1). Additionally, six themes were derived from the included PREMs: (1) organization, (2) treatment by healthcare providers, (3) expertise of healthcare providers, (4) patient empowerment, (5) end of treatment process, and (6) outpatient clinic.

**Table 1:** Included patient-reported experience measures (PREMs)

Included PREMs	Items extracted	Items in item bank
Consumer Quality Index (CQI) [10] (59 items)	23	16
Picker questionnaire [11] (22 items)	19	13
FACE-Q head and neck [12] (103 items)	25	8
PREM VBHC head and neck [13] (35 items)	24	10
EORTC-INFO25 [14] (25 items)	22	1
EORTC-COMU26 [15] (26 items)	20	6
Intern evaluation outpatient clinic (20 items) [16]	8	2
Formulated extra items	19	20
Total	160	76

### 2. Qualitative analyses of patient journey: focus group

In the focus group, the patient journey was discussed with head and neck cancer patients and healthcare professionals. Two key points in the full patient journey were visualized (the diagnostic stage and the follow-up phase), and additional contact moments were added. Six themes were derived from this qualitative analysis: (1) “Communication,” (2) “Information,” (3) “Shared decision-making,” (4) “Provided care,” (5) “Environment,” and (6) “Technology.” The topics considered important by the focus-group participants in the first phase are included in Fig. 2 and in the second phase in Fig. 3.

### 3. Combining themes from literature and themes from qualitative analysis

After comparing our themes resulting from the focus group with those derived from the literature, we proposed eight final themes: (1) organization, (2) competence of healthcare professionals, (3) communication, (4) information and services, (5) patient empowerment, (6) continuity of provided care, (7) environment, and (8) technology (Table 2). Further, during the qualitative analysis, 19 additional items were formulated that were not covered in the 141 items that were identified in the existing PREMs.

**Table 2:** Final PREM-item bank

Themes	Description	Items	Original questionnaire
1. Organization	Experience with the practical organization of healthcare. For example: time to consultation, accessibility of care, and waiting times	1. Were you satisfied with the time between the diagnostic tests and the diagnosis?	[10]
		2. Were you satisfied with the waiting time between the first consultation and the diagnosis?	-
		3. Did you like it that (if possible) several appointments for examination and/or treatment were scheduled on one day?	[10]
		4. Was your medical doctor available when you had questions or problems?	[12]
		5. Were the administrative staff friendly when you had questions or problems?	[11]
		6. Were you satisfied with the time between the consultation where treatment options were explained and the start of your treatment?	[16]
		7. Was the guidance of a volunteer on the first day in the hospital of added value?	[13]
2. Competence of healthcare professionals	Experience with the professionalism and expertise of healthcare professionals in order to provide optimal care	1. Did you trust your medical doctor?	[11]
		2. Did you trust the supportive care professionals at the outpatient clinic, such as speech therapists, physiotherapists, and social workers?	[11]
		3. Did your medical doctor have a professional attitude?	[12]
		4. Did your medical doctor consult other doctors or refer you if additional expertise was needed?	[10]
		5. Did your medical doctor or other healthcare providers read your file carefully?	[10]
		6. Did your medical doctor take action in response to your complaints (e.g., by prescribing medication for pain)?	[13]
3. Communication	Experience of the communication between healthcare professionals and patient and the social and communicative skills of the healthcare professionals	1. Did your medical doctor listen to you and understand your concerns?	[12]
		2. Did your medical doctor answer all your questions?	[12]
		3. Did your medical doctor put you at ease?	[12]
		4. If you had questions for other professionals at the outpatient clinic, did you get answers that you could understand?	[11]
		5. Was there enough time to talk about your illness or problems with the medical doctor or other professionals at the outpatient clinic?	[11]
		6. Were you satisfied with the communication between you and the specialist nurse?	[15]
		7. Did your medical doctor speak to you in a way you could understand?	[12]
		8. Did you feel free to ask questions to healthcare providers at the outpatient clinic?	[15]
		9. Was there mutual trust between you and your healthcare providers?	[15]
		10. Did your doctor discuss the answers you filled in on the health questionnaires with you?	-
		11. Did your healthcare provider seem honest to you?	[15]
		12. Did your healthcare provider try to understand your current situation?	[15]
		13. Did your medical doctor discuss the health problems that bother you the most?	[13]
		14. Did your healthcare providers pay enough attention to your wishes?	[10]
		15. Were you properly assisted by telephone by the secretariat or desk employee?	-
4. Information and services	Experience with the provided information so that patients are able to optimally communicate with their healthcare professionals	1. Did your medical doctor clearly explain what will happen during treatment?	[14]
		2. Were you satisfied with the information that you received about unexpected problems that could occur during treatment?	[10]
		3. Were you satisfied with the information that you received about possible complaints that could occur after treatment?	[10]
		4. Was it clear to you that you had cancer during treatment?	-
		5. Were you encouraged to take someone with you to the consultation where your treatment options discussed?	-
		6. Did you receive information about the existence of a patient organization?	[10]
		7. Was it clear for you with whom in the hospital you could discuss questions or problems once the treatment was finished?	[10]
		8. Was the provided written information about the diagnostic tests and treatments clear?	[10]
		9. Were you asked the way(s) you preferred to receive information?	-
		10. When you had to wait for your consultation, was it clear how long the waiting time was going to be?	[11]
		11. Did the letter you received at home clearly explain what the day at the hospital would be like?	[16]
		12. Did you receive information about the effects of smoking and alcohol on your illness?	[10]
5. Patient empowerment	Experience with the opportunity to participate in shared decision making, which will enhance patient empowerment and autonomy for the patient	1. Did you feel better prepared for the consultation with your medical doctor by filling in the health questionnaires?	[13]
		2. Did your medical doctor help you to decide what was best for you?	[12]
		3. Did your medical doctor treat you as an equal?	[15]
		4. Were you able to participate in decisions about your diagnostic tests or treatment?	[11]
		5. Were your family or friends able to think along and discuss your diagnostic tests or treatment?	[11]
		6. Did you have enough information to make a choice for treatment?	-
		7. Did someone clearly explain the advantages and disadvantages of the treatment to you?	[11]
		8. Did you have enough time to let the information you received sink in?	-
6. Continuity of provided care	Experience with the continuity of care (before or after treatment), when more medical specialties were involved	1. Did you have a permanent contact person to arrange your appointments?	[10]
		2. Did you see the same healthcare providers during your examinations and treatments (when possible)?	[10]
		3. Did you have a consultation with a specialist nurse after your diagnosis was confirmed by the medical doctor/breaking bad news?	[10]
		4. Did your healthcare provider, besides your illness, also look at your overall health?	-
		5. Was the necessary care and additional help for the home situation arranged in time?	[10]
		6. Was it clear who to contact with questions?	-
		7. Was there a healthcare provider in this hospital that you could call 24 h a day?	[10]
		8. Was there someone at the outpatient clinic you could talk to about your problems and fears?	[11]
		9. Were there moments when healthcare providers at the outpatient clinic told you contrary things, which left you confused?	[11]
		10. Did the healthcare providers involved in your care worked well together at the outpatient clinic?	[12]
7. Environment	Experience with the ambiance and decor of the outpatient clinic, including attention to privacy	1. Did you like the atmosphere in the waiting room?	-
		2. Were there drinks available in the waiting room?	-
		3. Did you like the temperature in the waiting room?	-
		4. Was your privacy handled well at the outpatient clinic?	-
		5. Was the route to the toilets at the outpatient clinic well marked?	-
		6. Were the toilets in the outpatient clinic clean?	-
		7. Was there enough space in the waiting room?	-
		8. Were you called in by name by the medical doctor?	-
8. Technology	Experience with the technology that is imbedded in the regular care. For example: experience with the use of an electronic patient reported outcome system	1. Was the help of the volunteer with the health questionnaires on the first day in the hospital of added value?	[13]
		2. Was the explanation by the specialist nurse about the health questionnaires of added value?	[13]
		3. Were you able to view your own results from the health questionnaires on the internet?	[13]
		4. Was it clear to you how you needed to register at the digital check-in point at the outpatient clinic?	-
		5. Do you have any comments or tips about our way of working with the health questionnaires ?	[13]
		6. Are you satisfied with the time it took you to complete the health questionnaires ?	[13]
Other	General experience	1. How would you rate your visit to the outpatient clinic?	[11]
		2. Would you recommend the department to others?	-
		3. Did you feel any topics were overlooked during the check-up visit to your medical doctor?	[13]
		4. Would you like to tell us anything else about the outpatient clinic? This can be positive or negative.	[11]

Health questionnaires = patient-reported outcome measures (PROMs)

### 4 & 5. Mapping and prioritizing

Subsequently, 160 items were mapped within the eight themes. Following this, 7 (ex-)patients and 11 healthcare professionals participated in prioritizing the items within each theme. The top-five items selected by the two groups, from each of the smaller themes (1, 2, 5, 7, and 8), and the top-ten from the larger themes (3, 4, and 6) were included in the item-bank (*n* = 57). In most cases, the prioritizations of the (ex-)patients and healthcare professionals were similar: 72% of the selected items were in the top five/ten of both groups, and 25% of the selected items were in the top five/ten of one group and in the top 11/16 respectively of the other group (Δ < 6 places). Only two items were selected in which the prioritization of (ex-)patients and healthcare professionals differed widely: “Were you satisfied with the communication between you and the specialist nurse?” (theme 3) was put in fifth place by the (ex-)patients but only 24th by the healthcare professionals. Further, “Did you receive information about the effects of smoking and alcohol on your illness?” (theme 4) was placed 11th and 22nd, respectively.

### 6. Qualitative analysis open comments

Eventually, the open comments were evaluated and discussed. An additional 19 items were added to the PREM-item bank after consensus of all three researchers. In total, 76 items were included in the final PREM-item bank (Table 2).

## Discussion

To the best of our knowledge, there is currently no patient-reported experience measure (PREM) item bank applicable to cancer care at an outpatient clinic. In this mixed-method study, we proposed a PREM-item bank that can be used to dynamically evaluate the provided care at a cancer outpatient clinic. Through the discussion of a patient journey with patients and professionals, which assessed the key contact moments of care during the intake and diagnostic phase and the follow-up phase, eight themes were derived. Together with patients and healthcare professionals, the most important items from existing PREMs were chosen and missing relevant items were added. Eventually, an item bank with 76 items was formed (Table 2).

Since the introduction of value-based healthcare (VBHC) in 2006, there has been increasing interest in the patient’s perspective on healthcare [17, 18]. Both the perspective of patients on their own functioning (PROMs) and their experiences with the provided care (PREMs) are seen as important instruments for quality improvement [3]. As the World Health Organization (WHO) notes, person-centered care is a key component of providing high-quality healthcare [19]. Furthermore, patient-reported experiences are associated with higher patient safety and clinical effectiveness [7, 20].

To date, most PREMs that are used are generic so that results can be compared between departments and hospitals (e.g., the patient experience monitor (PEM) [5]). Although the PEM is used in all academic university hospitals in the Netherlands, it can be difficult to pinpoint concrete actions to improve the care quality in specific departments based on the results of this generic PREM. Some of the evaluated items are outside the control of a department, such as how parking is experienced. The review by Gleeson et al. similarly noted the problem of a lack of specificity in national surveys, and that staff often thought that such data were not applicable or relevant to their daily work [8].

Furthermore, there are problems with the PEM in that there appears to be a ceiling effect: the overall experience is often rated as good (score of 8 out of 10) in all participating university hospitals [21]. This makes it difficult to further improve healthcare based on these already good scores. While we acknowledge the importance of this generic measurement for benchmarking experiences with the provided care, our PREM-item bank can complement this generic measurement with a department-specific measurement, in which the care delivered by a specific department can be evaluated. Each department has its own care pathways and may provide care in ways that are not seen in other departments (e.g., the use of volunteers on the first day).

In particular, the use of VBHC during consultations and remote care is relatively a new aspect of healthcare, and the ways these are provided often differ between departments. For patients to actively take part in VBHC, they have to contribute by completing questionnaires or using a web application. Since we can only request this of patients, it is particularly important that we continuously assess their experiences. On one hand, we want to identify areas where we can further improve the care we provide while, on the other hand, we want to know what patients already value. From an earlier study in which we used a department-specific PREM, we learned that patients did not see completing these PROMs as a burden, and, further, that filling in and discussing PROMs during consultations enhanced patient empowerment [13]. These appreciated aspects can be used to convince other stakeholders that VBHC leads to quality improvements from the patient’s perspective [22]. Although quality improvements based on PREM data have often been described, only a few studies report a quantitative impact as a result of using PREM-data [3]. It is our hypothesis that with the introduction of this dynamic manner of collecting department-specific PREM-data, identifying potential quality improvements will be more straightforward and applicable for a specific care pathway. When items are given the highest possible rating or it is not possible to further improve an aspect, PREM items could be changed in order to focus on another aspect of care. In this way, patients’ experiences will be able to optimally contribute to improving the quality of various aspects of healthcare. In the future, we intend to evaluate the impact of using aggregated PREM-data on patient-reported outcomes and experiences.

### Strengths and limitations

A strength of this study is its mixed-method design. Furthermore, inputs from both patients and healthcare professionals were taken into account when forming the PREM-item bank. Both groups prioritized similar items that they considered important to include in the PREM-item bank. This item bank could be used at any oncology department worldwide, and additional items can be added to enable the constantly evolving healthcare to be structurally evaluated. The questions were phrased in an easily understandable way so that patients with low health literacy could participate. A limitation is that our study only included head and neck cancer patients and healthcare professionals who were working in this area. Nevertheless, to make this item bank applicable to all cancer departments, we included both generic and cancer-specific PREMs based on our explorative literature search. Another limitation is that the literature search was explorative, limited to known PREMs, meaning that some relevant PREMs might have been overlooked. Furthermore, only PREMs validated in the Dutch language were included. In this phase, the item bank is not psychometrically validated, and we added non-validated items from our focus group analysis. However, this is the first step and validation will be performed in the upcoming paper.

### Future perspectives

In order to evaluate the VBHC offered in various oncology departments, we intend, in co-creation with other departments and patients, to develop a PREM-VBHC that will be structurally embedded in the provided care. We aim for the associated questionnaire to be limited to approximately 15 fixed items and 5 dynamic items. In this way, oncology departments can be compared, while department-specific care can be dynamically evaluated. The PREM-VBHC will be part of our electronic patient-reported outcome system so it will not increase the workload of healthcare professionals during their daily practice, which has been reported as a barrier to the use of PREMs [22]. The PREM-VBHC results will not be used during consultations as this could affect patients’ honesty [22]. Rather, the aggregated data will be discussed during regular meetings with all involved healthcare professionals following a plan-do-study-act cycle. Ideally, the aggregated PREM data will be complemented with aggregated PROM results. Although the use of aggregated PROMs is still in its infancy, their combination with PREMs for quality improvement seems promising [23]. The PREM-item bank can additionally be used as a basis when a specific topic needs to be addressed (e.g., acute care).

## Conclusions

In collaboration with patients and healthcare professionals, we have developed a bank of patient-reported experience measure (PREM) items to evaluate patients’ experiences while receiving cancer care in an outpatient clinic. This PREM-item bank is the first step to dynamically assess the quality of cancer care provided in an outpatient setting. The questions were formulated in straightforward language so that patients with limited health literacy could understand them. We will use the PREM-item bank to develop a PREM that is purposefully designed to evaluate the value-based healthcare that is offered in various oncology departments in our institute and could be used elsewhere.

### Supplementary information

ESM 1 (DOCX 47 kb)

## Data Availability

Data sharing is not applicable to this article as no datasets were generated or analyzed during the current study.

## Abbreviations

PREM: Patient-reported experience measure
PROM: Patient-reported outcome measure
VBHC: Value-based healthcare
CQI: Consumer Quality Index
PEM: Picker experience measure
EORTC-INFO25: EORTC information module
EORTC-COMU26: EORTC communication module
WHO: World Health Organization
