# Supplementary material for: The first steps in the development of a cancer-specific patient-reported experience measure item bank (PREM-item bank): towards dynamic evaluation of experiences
Source: Support Care Cancer. 2024 Jan 12;32(2):100. doi: 10.1007/s00520-023-08266-5 (PMC10786971; doi:10.1007/s00520-023-08266-5)
Supplement: Supplementary file 1 — (DOCX 47 kb) [file 520_2023_8266_MOESM1_ESM.docx]

**Thema 1 ‘organisatie in Erasmus MC’**

*Hoe wordt de organisatie van de zorg, inclusief de snelheid, toegankelijkheid zorgverleners, de opvang van nieuwe patiënten en de wachttijd op de polikliniek ervaren?*

1. Bent u tevreden over de tijd die er zat tussen het eerste onderzoek en de uitslag van uw diagnose (het vaststellen van uw ziekte)?

- **Ja**, ik ben tevreden over de tijd tussen het eerste onderzoek en mijn diagnose
- **Nee**, de tijd tussen het eerste onderzoek en mijn diagnose was **langer dan ik wilde**
- **Nee**, ik ben **erg ontevreden** over de tijd tussen het eerste onderzoek en mijn diagnose
- **Weet ik niet meer**

1. Bent u tevreden over de wachttijd tot uw eerste polikliekbezoek bij ons in het Erasmus MC Kankerinstituut?

- **Ja**, ik ben tevreden over de wachttijd tot mijn eerste polikliniekbezoek
- **Nee**, de wachttijd tot mijn eerste polikliniekbezoek was **langer dan ik wilde**
- **Nee**, ik ben **erg ontevreden** over de wachttijd tot mijn eerste polikliniekbezoek
- **Weet ik niet meer**

1. Vond u het prettig dat (indien mogelijk) meerdere afspraken voor onderzoek en/of behandeling op 1 dag werden gepland?

- **Ja**, ik vond het prettig dat meerdere afspraken op 1 dag werden gepland
- **Soms** vond ik het prettig dat meerdere afspraken op 1 dag werden gepland
- **Nee**, ik vond het niet prettig dat meerdere afspraken op 1 dag werden gepland
- **Weet ik niet meer**

1. Vond u dat de hoofd-hals chirurg beschikbaar was als u vragen of problemen had?

- **Ja**, de hoofd-hals chirurg was beschikbaar voor vragen of problemen
- **Soms** was de hoofd-hals chirurg beschikbaar voor vragen of problemen
- **Nee**, de hoofd-hals chirurg was niet beschikbaar voor vragen of problemen
- **Weet ik niet meer**

1. Vond u het administratief personeel vriendelijk als u vragen of problemen had?

- **Ja**, het administratief personeel was vriendelijk als ik vragen of problemen had
- **Soms** was het administratief personeel vriendelijk als ik vragen of problemen had
- **Nee**, het administratief personeel was niet vriendelijk als ik vragen of problemen had
- **Weet ik niet meer**

1. Bent u tevreden over de tijd die er zat tussen het gesprek waarin uw behandelplan werd voorgesteld en de start van uw behandeling?

- **Ja**, ik ben tevreden over de tijd tussen mijn behandelplan-gesprek en start behandeling
- **Nee**, de tijd tussen mijn behandelplan-gesprek en start behandeling was **langer dan ik wilde**
- **Nee**, ik ben **erg ontevreden** over de tijd tussen mijn behandelplan-gesprek en start behandeling
- **Weet ik niet meer**

1. Vond u de begeleiding van een vrijwilliger op de eerste dag in het ziekenhuis van toegevoegde waarde?

- **Ja**, ik vond de begeleiding van de vrijwilliger van toegevoegde waarde
- **Soms** vond ik de begeleiding van de vrijwilliger van toegevoegde waarde
- **Nee**, ik vond de begeleiding van de vrijwilliger niet van toegevoegde waarde
- **Weet ik niet meer**

**Thema 2 ‘deskundigheid zorgverleners’**

*Dit gaat over de professionaliteit en de ter zake kundigheid van zorgverleners om optimale patiëntenzorg te kunnen verlenen.*

1. Had u vertrouwen in de hoofd-hals chirurg?

- **Ja**, ik had vertrouwen in de hoofd-hals chirurg
- Ik had **minder vertrouwen** in de hoofd-hals chirurg dan ik wilde
- **Nee,** ik had geen vertrouwen in de hoofd-hals chirurg
- Ik had **geen afspraak** met de hoofd-hals chirurg
- Weet ik niet meer

1. Had u vertrouwen in de ondersteunende zorgverleners op de polikliniek, zoals logopedie, fysiotherapie, maatschappelijk werk?

- Ja, ik had vertrouwen in de ondersteunende zorgverleners
- Ik had minder vertrouwen in de ondersteunende zorgverleners
- Nee, ik had geen vertrouwen in de ondersteunende zorgverleners
- Ik had **geen afspraak met** de ondersteunende zorgverleners

1. Vond u dat de hoofd-hals chirurg een professionele werkhouding had?

- **Ja**, ik vond dat de hoofd-hals chirurg een professionele werkhouding had
- **Soms** vond ik dat de hoofd-hals chirurg een professionele werkhouding had
- **Nee** , ik vond dat de hoofd-hals chirurg geen professionele werkhouding had
- Ik had **geen afspraak** met de hoofd-hals chirurg
- **Geen mening**

1. Overlegde uw hoofd-hals chirurg met andere artsen of werd u doorverwezen als er extra deskundigheid nodig was?

- **Ja**, mijn hoofd-hals chirurg heeft toen dit nodig was overleg gehad met andere zorgverleners of mij naar hen **doorverwezen**
- **Nee**, mijn hoofd-hals chirurg heeft toen dit nodig was geen overleg gehad of mij doorverwezen naar andere zorgverleners
- Er was **geen overleg nodig** met andere zorgverleners
- **Weet ik niet meer**

1. Hadden de artsen of andere zorgverleners die u sprak uw dossier goed gelezen?

- **Ja,** ze hadden mijn dossier goed genoeg gelezen
- Ze hadden mijn dossier wel gelezen, maar **niet genoeg**
- **Nee,** ze hadden **weinig of niets** van mijn dossier gelezen
- Ik heb **geen arts** of andere zorgverleners **gesproken**
- **Weet ik niet meer**

1. Mijn arts ondernam actie naar aanleiding van mijn klachten (bijv. door het voorschrijven van medicatie bij pijn)

- **Ja**, mijn arts ondernam **actie** naar aanleiding van mijn klachten
- **Soms** ondernam mijn arts actie naar aanleiding van mijn klachten, niet vaak genoeg
- **Nee**, mijn arts ondernam **geen actie** naar aanleiding van mijn klachten
- **Ik heb geen klachten gehad** waarop actie ondernomen moest worden
- **Weet ik niet meer**

**Thema 3 ‘communicatie en bejegening’**

*Gaat over de communicatie binnen de relatie van de patiënt met de zorgprofessional en de sociale en communicatieve vaardigheden die zorgprofessionals tonen waarin de patiënt wordt gezien als ‘partner’ in de zorg.*

1. Vond u dat de hoofd-hals chirurg naar u luisterde en begrip toonde voor uw zorgen?

- **Ja**, de hoofd-hals chirurg luisterde naar mij en toonde begrip voor mijn zorgen
- **Meestal** luisterde de hoofd-hals chirurg naar mij en toonde begrip voor mijn zorgen, maar **niet altijd**
- **Nee**, de hoofd-hals chirurg luisterde niet naar mij en toonde geen begrip voor mijn zorgen
- **Weet** ik **niet meer**

1. Vond u dat de hoofd-hals chirurg al uw vragen beantwoordde?

- **Ja**, de hoofd-hals chirurg heeft al mijn vragen beantwoord
- **Meestal** heeft de hoofd-hals chirurg mijn vragen beantwoord, maar **niet allemaal**
- **Nee**, de hoofd-hals chirurg heeft mijn vragen niet beantwoord
- **Weet** ik **niet meer**

1. Vond u dat de hoofd-hals chirurg u op uw gemak stelde?

- **Ja**, de hoofd-hals chirurg heeft mij op mijn gemak gesteld
- **Meestal** heeft de hoofd-hals chirurg mij op mijn gemak gesteld, maar **niet altijd**
- **Nee**, de hoofd-hals chirurg heeft mij niet op mijn gemak gesteld
- **Weet** ik **niet meer**

1. Als u vragen had voor de andere zorgverleners op de polikliniek, kreeg u dan antwoorden die u kon begrijpen?

- **Ja**, de antwoorden van andere zorgverleners waren te begrijpen
- **Meestal** waren de antwoorden van andere zorgverleners te begrijpen, **soms niet**
- **Nee**, de antwoorden van andere zorgverleners waren **niet** te begrijpen
- Ik had **geen vragen voor andere zorgverleners**
- **Weet** ik **niet meer**

1. Was er genoeg tijd om met de arts of andere zorgverleners op de polikliniek te praten over uw ziekte of probleem?

- **Ja**, er was **genoeg tijd** om te praten over mijn ziekte of probleem
- **Meestal** was er tijd, maar **ik had meer tijd nodig** om te praten over mijn ziekte of probleem
- **Nee**, er was **geen tijd** om te praten over mijn ziekte of probleem
- Ik had **geen behoefte om te praten** over mijn ziekte of probleem
- **Weet** ik **niet meer**

1. Was u tevreden over de communicatie tussen u en de verpleegkundig consulent?

- **Ja**, ik was tevreden over de communicatie tussen mij en de verpleegkundig consulent
- **Meestal** was ik tevreden over de communicatie tussen mij en de verpleegkundig consulent
- **Nee**, ik was niet tevreden over de communicatie tussen mij en de verpleegkundig consulent
- **Weet** ik **niet meer**

1. Toen u in gesprek was met uw chirurg sprak deze met u op een manier die voor u begrijpelijk was?

- **Ja**, de chirurg sprak begrijpelijk
- **Meestal** sprak de chirurg begrijpelijk, **soms niet**
- **Nee**, de chirurg sprak niet begrijpelijk
- **Weet** ik **niet meer**

1. Voelde u zich op de polikliniek vrij genoeg uw vragen te stellen aan zorgverleners in het algemeen?

- **Ja**, ik voelde me vrij genoeg vragen te stellen
- **Meestal** voelde ik me vrij genoeg vragen te stellen, **soms niet**
- **Nee**, ik voelde me **niet vrij genoeg** vragen te stellen
- Ik had **geen vragen**
- **Weet** ik **niet meer**

1. Was er sprake van wederzijds vertrouwen tussen u en uw zorgverlener(s)?

- **Ja**, er was **wederzijds vertrouwen** tussen mij en mijn zorgverleners
- **Meestal** was er wederzijds vertrouwen met mijn zorgverleners, **soms niet**
- **Nee**, er was **geen wederzijds vertrouwen** tussen mij en mijn zorgverleners
- **Weet** ik **niet meer**

1. Heeft uw arts de door u ingevulde antwoorden op de gezondheids-vragenlijsten met u besproken?

- **Ja**, mijn arts heeft mijn antwoorden besproken
- **Meestal** heeft mij arts mijn antwoorden besproken, **soms niet**
- **Nee**, mijn arts heeft mijn antwoorden **niet besproken**
- Ik heb **geen** gezondheidsvragenlijsten **ingevuld**
- **Weet** ik **niet meer**

1. Kwam uw zorgverlener eerlijk op u over?

- **Ja**, mijn zorgverlener kwam eerlijk op mij over
- **Meestal,**  kwam mijn zorgverlener eerlijk op mij over, **soms niet**
- **Nee**, mijn zorgverlener kwam niet eerlijk op mij over
- **Weet** ik **niet meer**

1. Probeerde uw zorgverlener uw situatie van dit moment te begrijpen?

- **Ja**, mijn arts probeerde mijn situatie te begrijpen
- **Meestal** probeerde mijn arts mijn huidige situatie te begrijpen, **soms niet**
- **Nee**, mijn arts probeerde onvoldoende mijn situatie te begrijpen
- **Weet** ik **niet meer**

13. Heeft de hoofd-hals chirurg de gezondheidsklachten waar u het meest last van heeft met u besproken?

- **Ja**, mijn arts heeft alle gezondheidsklachten waar ik last van had met mij besproken
- Mijn arts heeft de meeste gezondheidsklachten waar ik last van heb met mij besproken, **niet allemaal**
- **Nee**, mijn arts heeft de gezondheidsklachten waar ik last van had **niet besproken**
- **Weet** ik **niet meer**

1. Vond u dat de zorgverleners aandacht hadden voor uw wensen?

- **Ja**, de zorgverleners hadden aandacht voor mijn wensen
- Ik vond dat de zorgverleners **meestal** aandacht hadden voor mijn wensen
- **Nee**, ik vond dat de zorgverleners geen aandacht hadden voor mijn wensen
- **Weet** ik **niet meer**

1. Werd u telefonisch goed geholpen door secretariaat of baliemedewerker?

- **Ja**, ik werd telefonisch goed geholpen
- **Meestal** werd ik telefonisch goed geholpen, **soms niet**
- **Nee**, ik werd telefonisch niet goed geholpen
- **Weet** ik **niet meer**
- Ik heb **geen telefonisch contact** gehad met administratief personeel

**Thema 4 ‘informatie / voorzieningen’**

*Dit gaat over het instrueren en het optimaal voorzien van informatie aan patiënten die nodig is om optimaal te kunnen communiceren met zorgprofessionals.*

1. Heeft de hoofd-hals chirurg u duidelijk uitgelegd hoe de behandeling zal gaan?

- **Ja**, de chirurg heeft de behandeling **duidelijk** uitgelegd
- De chirurg heeft de behandeling uitgelegd, maar **niet altijd duidelijk**
- **Nee**, de chirurg heeft de behandeling **niet duidelijk** uitgelegd
- Dat was **niet nodig,** want ik kreeg geen behandeling
- **Weet** ik **niet meer**

1. Hoe tevreden was u over de informatie die u hebt ontvangen over onverwachte problemen die konden ontstaan tijdens de behandeling?

- Ik was **tevreden** met de informatie over onverwachte problemen
- Ik was **een beetje tevreden** met de informatie over onverwachte problemen
- Ik was **niet tevreden** met de informatie over onverwachte problemen
- Ik kreeg **geen informatie** over onverwachte problemen
- **Weet** ik **niet meer**

1. Hoe tevreden was u over de informatie die u hebt ontvangen over mogelijke klachten na behandeling?

- Ik was **tevreden** met de informatie over mogelijke klachten na behandeling
- Ik was **een beetje tevreden** met de informatie over mogelijke klachten na behandeling
- Ik was **niet tevreden** met de informatie over mogelijke klachten na behandeling
- Ik kreeg **geen informatie** over mogelijke klachten na behandeling
- **Weet** ik **niet meer**

1. Was het u tijdens de uitleg over de behandeling duidelijk dat u hoofd-hals kanker heeft?

- **Ja**, dat was mij gelijk duidelijk
- Dat was mij duidelijk maar pas **op** **een later moment**
- **Nee** dat was mij pas heel laat duidelijk
- **Weet** ik **niet meer**

1. Bent u aangemoedigd samen met iemand te komen naar het gesprek waarin uw behandelvoorstel werd besproken?

- Ik ben **aangemoedigd** samen naar het gesprek te komen
- Ik ben **aangemoedigd** maar vond het niet nodig om samen met iemand naar het gesprek te komen
- Ik ben **niet aangemoedigd**, maar ik kwam zelf al samen met iemand naar het gesprek
- Ik ben **niet aangemoedigd**, maar ik had dat wel prettig gevonden
- **Weet** ik **niet meer**

1. Kreeg u informatie over de patiëntenorganisatie?

- **Ja**, ik kreeg informatie over de patiëntenorganisatie
- **Nee**, ik kreeg geen informatie over de patiëntenorganisatie , maar ik **had** dat **wel gewenst**
- **Nee**, ik kreeg geen informatie over de patiëntenorganisatie, maar ik **wenste** deze informatie ook **niet**
- **Weet** ik **niet meer**

1. Wist u met wie u vragen of problemen kon bespreken in het ziekenhuis toen uw behandeling klaar was?

- **Ja**, ik wist na mijn behandeling met wie ik in het ziekenhuis mijn vragen of problemen kon bespreken
- Ik wist **niet altijd** na mijn behandeling met wie ik in het ziekenhuis mijn vragen of problemen kon bespreken
- **Nee**, ik wist na mijn behandeling niet met wie ik in het ziekenhuis mijn vragen of problemen kon bespreken
- **Weet** ik **niet meer**

1. Vond u de schriftelijke informatie over uw onderzoeken en behandeling duidelijk?

- **Ja**, ik vond de schriftelijke informatie duidelijk
- **Meestal** vond ik de schriftelijke informatie duidelijk, **niet altijd**
- **Nee**, ik vond de schriftelijk informatie niet duidelijk
- Ik heb **geen schriftelijke informatie** gekregen
- **Weet** ik **niet meer**

1. Werd aan u gevraagd hoe u graag informatie wilde ontvangen? U mag meerdere antwoorden aankruisen

- Ja, dit werd gevraagd en ik wil dat informatie **door een persoon** wordt uitgelegd
- Ja, dit werd gevraagd en ik wil informatie graag **op papier** ontvangen
- Ja, dit werd gevraagd en ik wil informatie graag **digitaal** (via e-mail of het patiënten portaal) ontvangen
- Ja, dit werd gevraagd en ik heb **geen voorkeur** hoe ik informatie ontvang
- Nee, dit werd niet gevraagd

1. Als u moest wachten in de wachtkamer, was het u duidelijk hoe lang u moest wachten?

- **Ja,** dat was duidelijk voor mij
- **Meestal** is dat duidelijk voor mij, **niet altijd**
- **Nee,** het was niet duidelijk voor mij maar dat vond ik **niet erg**
- **Nee,** het was niet duidelijk voor mij, ik had het **wel graag willen weten**
- **Weet** ik **niet meer**

1. Stond in de brief die u thuis gestuurd kreeg duidelijk uitgelegd hoe de dag in het ziekenhuis zou zijn?

- **Ja**, dat is **duidelijk** uitgelegd
- **Sommige dingen** zijn **niet duidelijk** uitgelegd
- **Nee**, dat is **niet duidelijk** uitgelegd
- Ik heb **geen brief** gekregen
- **Weet** ik **niet meer**

1. Heeft u informatie gekregen over de gevolgen van roken en alcohol op uw ziekte?

- **Ja**, ik kreeg informatie over de gevolgen van roken en alcohol op mijn ziekte
- **Nee**, ik kreeg geen informatie over de gevolgen van roken en alcohol op mijn ziekte, dit had ik **wel gewenst**
- **Nee**, ik kreeg geen informatie over de gevolgen van roken en alcohol op mijn ziekte, maar ik **wenste** deze informatie ook **niet**
- **Weet** ik **niet meer**

**Thema 5 ‘patiënt emancipatie’**

*Wanneer er optimaal wordt gecommuniceerd tussen patiënt en zorgprofessional inclusief de best mogelijke informatie die voor dat moment en die patiënt geldt, kan gedeelde besluitvorming mogelijk worden gemaakt voor die mensen die dat kunnen en willen. Hierdoor ontstaat meer eigen regie en autonomie voor de patiënt.*

1. Voelde u zich door het invullen van de gezondheidsvragenlijsten beter voorbereid op het gesprek met uw arts?

- **Ja**, door het invullen van de vragenlijsten vóór het gesprek met mijn arts, voelde ik mij beter voorbereid
- **Meestal** voelde ik mij beter voorbereid op het gesprek met mijn arts door het invullen van de vragenlijsten, **niet altijd**
- **Nee**, door het invullen van de vragenlijsten vóór het gesprek met mijn arts, voelde ik mij **niet** beter voorbereid
- Ik heb **geen vragenlijsten ontvangen** vóór het gesprek met mijn arts
- **Weet** ik **niet meer**

1. Vond u dat de hoofd-hals chirurg u hielp om te beslissen wat het beste voor u was?

- **Ja**, de hoofd-hals chirurg hielp mij om te beslissen wat het beste voor mij was
- **Meestal** hielp de hoofd-hals chirurg mij om te beslissen wat het beste voor mij was, **niet altijd**
- **Nee**, de hoofd-hals chirurg hielp mij **niet** om te beslissen wat het beste voor mij was
- Ik heb **geen beslissingen** hoeven nemen met mijn hoofd-hals chirurg
- **Weet** ik **niet meer**

1. Behandelde uw hoofd-hals chirurg u als gelijke?

- **Ja**, de hoofd-hals chirurg behandelde mij als gelijke
- **Meestal** behandelde de hoofd-hals chirurg mij als gelijke, **niet altijd**
- **Nee**, de hoofd-hals chirurg behandelde mij **niet** als gelijke
- **Weet** ik **niet meer**

1. Kon u meebeslissen over uw behandeling of onderzoek?

- **Ja**, ik kon meebeslissen over mijn behandeling of onderzoek
- **Nee**, ik kon **niet** meebeslissen over mijn behandeling of onderzoek, maar ik **had dat graag gewild**
- **Nee**, ik kon **niet** meebeslissen over mijn behandeling of onderzoek, maar ik **wilde** dat ook **niet**
- Ik heb **geen beslissingen** hoeven nemen of onderzoek gehad
- **Weet** ik **niet meer**

1. Kon een familielid of naaste meedenken en meepraten over uw behandeling of onderzoek?
   - **Ja**, een familielid of naaste kon meedenken en meepraten
   - Een familielid of naaste kon meedenken en meepraten, **niet zo vaak als ik wilde**
   - **Nee**, een familielid of naaste kon **niet** meedenken en meepraten
   - Ik heb **geen familielid of naaste** die kunnen mee denken en meepraten
   - **Weet** ik **niet meer**
2. Vindt u dat u voldoende informatie had om de keuze voor uw behandeling te kunnen maken?
   - **Ja**, ik had voldoende informatie om mijn keuze te maken
   - **Meestal** ontving ik **voldoende** informatie om mijn keuze te maken, **niet altijd**
   - **Nee**, ik heb **onvoldoende** informatie ontvangen om mijn keuze te maken
   - Ik heb **geen keuze voor een behandeling gehad**
   - **Weet** ik **niet meer**
3. Heeft iemand u duidelijk uitgelegd wat de voordelen en nadelen van de behandeling zijn die u kreeg?

- **Ja**, de voordelen en nadelen zijn **duidelijk** uitgelegd
- **De voordelen en nadelen zijn** uitgelegd, niet allemaal
- **Nee**, de voordelen en nadelen zijn **niet duidelijk** uitgelegd
- Dat was **niet nodig,** want ik kreeg geen behandeling
- **Weet** ik **niet meer**

1. Vindt u dat u voldoende tijd kreeg om de ontvangen informatie tot u te laten doordringen?
   - **Ja**, ik heb **voldoende** tijd gekregen om de ontvangen informatie tot mij te laten doordringen.
   - Ik heb tijd gehad om de ontvangen informatie tot mij te laten doodringen, maar ik had **graag iets langer de tijd gehad**
   - **Nee**, ik heb **onvoldoende** tijd gekregen om de ontvangen informatie tot mij te laten doordringen
   - Ik heb **geen informatie gekregen**
   - **Weet** ik **niet meer**

**Thema 6 ‘zorg ondersteunend en afstemming zorg’**

*Dit gaat over de dienstverlening in de zorg voor of na de behandeling vanuit verschillende expertisegebieden inclusief een goede afstemming van de boodschap naar de patiënt vanuit die verschillende experts.*

1. Had u een vast contactpersoon voor het regelen van uw afspraken?

- **Ja**, ik had een **vast contactpersoon**
- Ik had een vast contact persoon maar kon deze **niet altijd** bereiken
- **Nee**, ik had geen vast contactpersoon
- **Weet ik niet meer**

1. Zag u (zoveel mogelijk) dezelfde zorgverleners tijdens uw onderzoeken en behandelingen?

- **Ja**, ik zag dezelfde zorgverleners tijdens mijn onderzoeken en behandeling
- **Meestal** heb ik dezelfde zorgverleners gezien tijdens mijn onderzoeken en behandeling, **niet altijd**
- **Nee**, ik zag niet dezelfde zorgverleners tijdens mijn onderzoeken en behandeling
- **Weet ik niet meer**

1. Kreeg u na een slecht-nieuwsgesprek meteen een gesprek met een verpleegkundig consulent?

- **Ja**, ik kreeg na mijn slecht-nieuwsgesprek **een gesprek** met een verpleegkundig consulent
- **Nee**, er werd **geen gesprek** met een verpleegkundig consulent, maar ik had daar ook **geen behoefte aan**.
- **Nee**, er werd **geen gesprek** met een verpleegkundig consulent, maar ik had dat **wel graag gewild**
- Ik heb **geen slecht-nieuwsgesprek** gehad
- **Weet ik niet meer**

1. Vond u dat uw zorgverlener niet alleen naar uw ziekte keek maar ook aandacht had voor uw totale gezondheid?

- **Ja**, de zorgverlener keek ook naar mijn totale gezondheid
- **Meestal** keek de zorgverlener ook naar mijn totale gezondheid, **niet altijd**
- **Nee**, de zorgverlener keek niet naar mijn totale gezondheid
- **Weet ik niet meer**

1. Was de benodigde zorg en hulp voor de thuissituatie op tijd geregeld?

- **Ja**, de benodigde zorg en hulp voor de thuissituatie was op tijd geregeld
- **Het meeste** van de benodigde zorg en hulp voor de thuissituatie was op tijd geregeld, **niet alles**
- **Nee**, de benodigde zorg en hulp voor de thuissituatie was niet op tijd geregeld
- **Weet ik niet meer**

1. Was het voor u duidelijk aan wie u op welk moment vragen kon stellen?

- **Ja**, het was duidelijk aan wie ik op welk moment vragen kon stellen
- **Meestal** was het duidelijk aan wie ik op welk moment vragen kon stellen, **niet altijd**
- **Nee**, het was niet duidelijk aan wie ik op welk moment vragen kon stellen
- **Weet ik niet meer**

1. Was er een zorgverlener in dit ziekenhuis die u 24 uur per dag kon bellen?

- **Ja**, er was een zorgverlener die ik 24 uur per dag kon bellen
- **Meestal** was er een zorgverlener die ik 24 uur per dag kon bellen, **niet altijd**
- **Nee**, er was geen zorgverlener die ik 24 uur per dag kon bellen
- **Weet ik niet meer**

1. Was er iemand op de polikliniek met wie u kon praten over problemen en angsten?

- **Ja**, er was iemand op de polikliniek met wie ik kon praten over problemen en angsten
- **Meestal** was er iemand op de polikliniek met wie ik kon praten over problemen en angsten, **niet altijd**
- **Nee**, er was niemand op de polikliniek met wie ik kon praten over problemen en angsten
- Ik had **geen problemen of angsten**
- **Weet ik niet meer**

1. Waren er momenten dat artsen of andere zorgverleners op de polikliniek u niet hetzelfde vertelden waardoor u in de war raakte?

- **Ja**, er waren **vaak** momenten dat ik in de war raakte
- **Soms** waren er momenten dat ik in de war raakte
- **Nee**, er waren geen momenten dat ik in de war raakte
- Er waren **geen momenten** dat zorgverleners mij niet hetzelfde vertelden
- **Weet ik niet meer**

1. Vond u dat alle betrokken zorgverleners goed samenwerkten op de polikliniek?

- **Ja**, ik vond dat de zorgverleners goed samenwerkten op de polikliniek
- **Meestal** vond ik dat de zorgverleners goed samenwerkten op de polikliniek, **niet altijd**
- **Nee**, ik vond dat de zorgverleners niet goed samenwerkten op de polikliniek

**Thema 7 ‘omgeving’**

*Hoe wordt de sfeer en de aankleding op de polikliniek ervaren inclusief de aandacht voor privacy in de ruimten?*

1. Vond u de sfeer in de wachtkamer prettig?

- **Ja**, ik vond de sfeer in de wachtkamer prettig
- **Meestal** vond ik de sfeer in de wachtkamer prettig, **niet altijd**
- **Nee**, ik vond de sfeer in de wachtkamer niet prettig
- **Weet ik niet meer**

1. Was er drinken in de wachtkamer beschikbaar?

- **Ja**, er was koffie, thee of water beschikbaar is in de wachtkamer
- **Nee**, er was geen drinken beschikbaar, maar ik heb het **niet gemist**
- **Nee**, er was geen drinken beschikbaar, ik heb dat wel **gemist**
- **Weet** ik **niet meer**

1. Vond u de temperatuur in de wachtkamer prettig

- **Ja**, ik vond de temperatuur in de wachtkamer prettig
- **Meestal** vond ik de temperatuur in de wachtkamer prettig, **niet altijd**
- **Nee**, ik vond het **te koud** in de wachtkamer
- **Nee**, ik vond het **te warm** in de wachtkamer
- **Weet ik niet meer**

1. Vond u dat er op de polikliniek goed met uw privacy werd omgegaan?

- **Ja**, ik vond dat er goed met mijn privacy werd omgegaan
- **Meestal** werd er goed met mijn privacy omgegaan, **niet altijd**
- **Nee**, ik vond dat er niet goed met mijn privacy werd omgegaan
- **Weet ik niet meer**

1. Vond u dat de route naar de toiletten op de polikliniek goed aangegeven?

- **Ja,** de route naar de toiletten op de polikliniek staat goed aangegeven
- **Nee,**  de route naar de toiletten op de polikliniek staat niet goed aangegeven
- Ik heb geen gebruik gemaakt van de toiletten op de polikliniek
- Weet ik niet meer

1. Vond u dat de toiletten op de polikliniek schoon waren?

- **Ja,** de toiletten op de polikliniek waren schoon
- **Meestal** waren de toiletten op de polikliniek schoon, **niet altijd**
- **Nee**, de toiletten op de polikliniek waren niet schoon
- Ik heb **geen gebruik gemaakt** van de toiletten op de poli
- **Weet** ik **niet meer**

1. Was er genoeg plek in de wachtkamer?

- **Ja**, er was vaak genoeg plek in de wachtkamer
- **Meestal** was er genoeg plek in de wachtkamer, **niet altijd**
- **Nee**, er was niet genoeg plek in de wachtkamer
- **Weet** ik **niet meer**

1. Werd u bij naam binnengeroepen door de arts?

- **Ja**, ik werd bij naam binnengeroepen door de arts en ik heb daar **geen problemen mee**
- **Ja**, ik werd bij naam binnengeroepen door de arts en ik heb daar **problemen mee**
- **Nee**, ik werd niet bij naam naar binnengeroepen
- **Weet** ik **niet meer**

**Thema 8 ‘technologie’**

*Dit gaat over technologie die wordt ingezet om optimale zorg en service te verlenen. Te denken valt aan werkwijze met de Zorgmonitor waardoor er een gericht gesprek in de spreekkamer plaatsvindt aan de hand van een dashboard, of de digitale aanmeldzuilen in het ziekenhuis.*

1. Vond u de hulp van de vrijwilliger op de eerste dag in het ziekenhuis van toegevoegde waarde bij het invullen van de gezondheidsvragenlijsten?

- **Ja**, ik vond de hulp bij het invullen van de Zorgmonitor van **toegevoegde waarde**
- **Meestal** vond ik de hulp bij het invullen van de Zorgmonitor van toegevoegde waarde, **niet altijd**
- **Nee**, ik vond de hulp niet van toegevoegde waarde
- Ik kreeg **geen hulp** van een vrijwilliger
- Ik heb de Zorgmonitor **thuis ingevuld**
- **Weet ik niet meer**

1. Vond u de uitleg van de verpleegkundig consulent over de gezondheidsvragenlijsten nuttig?

- **Ja**, ik vond de uitleg over de Zorgmonitor nuttig
- **Meestal** vond ik de uitleg over de Zorgmonitor nuttig, **niet altijd**
- **Nee**, ik vond de uitleg over de Zorgmonitor **niet** nuttig
- Ik kreeg **geen uitleg** over de Zorgmonitor van de verpleegkundig consulent
- **Weet ik niet meer**

1. Heeft u behoefte in de toekomst via internet uw eigen uitslagen van de gezondheidsvragenlijsten te kunnen bekijken?

- **Ja**, ik heb hier behoefte aan
- **Nee**, ik heb hier geen behoefte aan
- **Weet ik niet**

1. Was het aanmelden via de aanmeldzuil op de polikliniek KNO voor u duidelijk?

- **Ja**, ik vond het aanmelden via de aanmeldzuil op de polikliniek KNO **duidelijk**
- **Meestal** vond ik het aanmelden via de aanmeldzuil op de polikliniek KNO duidelijk**, niet altijd**
- **Nee**, ik vond het aanmelden via de aanmeldzuil op de polikliniek KNO **niet duidelijk**
- **Weet ik niet meer**

1. Heeft u opmerkingen of tips voor ons over onze werkwijze met de gezondheidsvragenlijsten?

- **Nee**, ik heb geen opmerkingen of tips over de werkwijze
- **Ja,** ik heb opmerkingen of tips over de werkwijze, namelijk…………………..

1. Bent u tevreden over de tijd die het u kostte de gezondheidsvragenlijsten vragenlijsten in te vullen?

- **Ja**, ik ben tevreden over de tijd die het me kostte
- **Nee**, de tijd die het mij kostte was **langer dan ik wilde**
- **Nee**, ik ben **erg ontevreden** over de tijd die het me kostte
- **Weet ik niet meer**

**‘Ervaring algemeen’**

1. Welk cijfer geeft u voor uw bezoek op de polikliniek?

0 betekent ‘Heel slecht’, 10 betekent ‘Heel goed’

1. Zou u de afdeling KNO-HH oncologie aan anderen aanraden?

[Net Promotor Score]

1. Heeft u onderwerpen gemist tijdens het controlebezoek bij de hoofd-hals chirurg?

[Ik heb de volgende onderwerpen gemist: open veld]

1. Wilt u nog iets anders vertellen over de polikliniek? Dit kan positief en negatief zijn.

[open veld]
